# The Earth’s magnetic field in Jerusalem during the Babylonian destruction: A unique reference for field behavior and an anchor for archaeomagnetic dating

**DOI:** 10.1371/journal.pone.0237029

**Published:** 2020-08-07

**Authors:** Yoav Vaknin, Ron Shaar, Yuval Gadot, Yiftah Shalev, Oded Lipschits, Erez Ben-Yosef

**Affiliations:** 1 Institute of Archaeology, Tel Aviv University, Tel Aviv, Israel; 2 Institute of Earth Sciences, The Hebrew University of Jerusalem, Jerusalem, Israel; 3 Israel Antiquities Authority, Jerusalem, Israel; Universita degli Studi di Milano, ITALY

## Abstract

Paleomagnetic analysis of archaeological materials is crucial for understanding the behavior of the geomagnetic field in the past. As it is often difficult to accurately date the acquisition of magnetic information recorded in archaeological materials, large age uncertainties and discrepancies are common in archaeomagnetic datasets, limiting the ability to use these data for geomagnetic modeling and archaeomagnetic dating. Here we present an accurately dated reconstruction of the intensity and direction of the field in Jerusalem in August, 586 BCE, the date of the city’s destruction by fire by the Babylonian army, which marks the end of the Iron Age in the Levant. We analyzed 54 floor segments, of unprecedented construction quality, unearthed within a large monumental structure that had served as an elite or public building and collapsed during the conflagration. From the reconstructed paleomagnetic directions, we conclude that the tilted floor segments had originally been part of the floor of the second story of the building and cooled after they had collapsed. This firmly connects the time of the magnetic acquisition to the date of the destruction. The relatively high field intensity, corresponding to virtual axial dipole moment (VADM) of 148.9 ± 3.9 ZAm^2^, accompanied by a geocentric axial dipole (GAD) inclination and a positive declination of 8.3°, suggests instability of the field during the 6^th^ century BCE and redefines the duration of the Levantine Iron Age Anomaly. The narrow dating of the geomagnetic reconstruction enabled us to constrain the age of other Iron Age finds and resolve a long archaeological and historical discussion regarding the role and dating of royal Judean stamped jar handles. This demonstrates how archaeomagnetic data derived from historically-dated destructions can serve as an anchor for archaeomagnetic dating and its particular potency for periods in which radiocarbon is not adequate for high resolution dating.

## Introduction

Archaeomagnetism, the application of paleomagnetic methods to archaeological materials, is interdisciplinary not only in its methods but also in its impact. Well-dated archaeological materials are a critical data source for geomagnetic secular variation models [1–6], which are used to explore the dynamic structure of Earth’s core [7, 8], the rates of cosmogenic isotope production in the atmosphere [9–11] and the possible effect of geomagnetism on climate [11–13]. Precise documentation of the ancient field also helps contextualize geomagnetic observations from the modern era, such as the evolution of the South Atlantic Anomaly [14, 15] and the ongoing decline in the field’s intensity [16–18]. In the archaeological research of the Levant, the growing body of archaeomagnetic data [19–21] enables an increasingly reliable dating method [22–24]. In Western Europe this dating method has proven to be especially useful during periods in which high resolution radiocarbon dating is not possible [25]. Archaeomagnetism can also provide a powerful tool for reconstructing site formation processes [26–29].

Archaeomagnetism is typically based on materials that were heated to high temperatures and acquired thermoremanent magnetization (TRM) during their cooling. The TRM is parallel and proportional to the field in which the material cooled and thus it enables indirect estimation of the direction and the intensity of the field at the time the material last cooled. There is an inherent difficulty in accurately dating the last heating event of archaeological materials that were repeatedly heated during their everyday use, such as kilns, ovens and hearths. Pottery, whose usage could have been up to several decades long, poses a similar difficulty since its heating event, during production, is detached from the archaeological context in which it was found. For these reasons, archaeomagnetic datasets sometimes consist of large age uncertainties, limiting the resolution of archaeomagnetic records. In contrast to conventional materials, substances that were heated during historically-dated destruction events represent a precise and well-defined discrete point in time. This unique type of information is especially critical for periods with fast changes in the field, such as “geomagnetic spikes” [30–32] and “archaeomagnetic jerks” (Gallet et al., 2003) and when conventional archaeological and radiocarbon dating lack the required resolution [33]. Here we focus on one such event that marks the end of the Iron Age in the Southern Levant–the Babylonian destruction of Jerusalem in August, 586 BCE [34]. The significance of this event extends beyond its historical context. From a geomagnetic perspective, 586 BCE was believed to follow a period of an intense high-field anomaly identified in the Near East [32, 35] and in Western Europe [5, 36, 37]. From a chronological viewpoint the destruction of Jerusalem in 586 BCE took place in the middle of a period characterized by a plateau in the radiocarbon calibration curve [38], posing difficulty in obtaining uncertainty of less than 200 years in standard radiocarbon dating. For example, S1 Fig displays the radiocarbon calibration curve of a synthetic radiocarbon age of 2485±25 Before Present (BP), which corresponds (by reverse calibration) to ca. 586 calibrated BC (calBC). The 95.4% confidence interval of the calibrated age spans over more than 250 years (771–517 calBC) and the 68.2% confidence intervals range over 216 years (756–545 calBC) [38, 39].

### Archaeological and historical context

**Fig 1:**
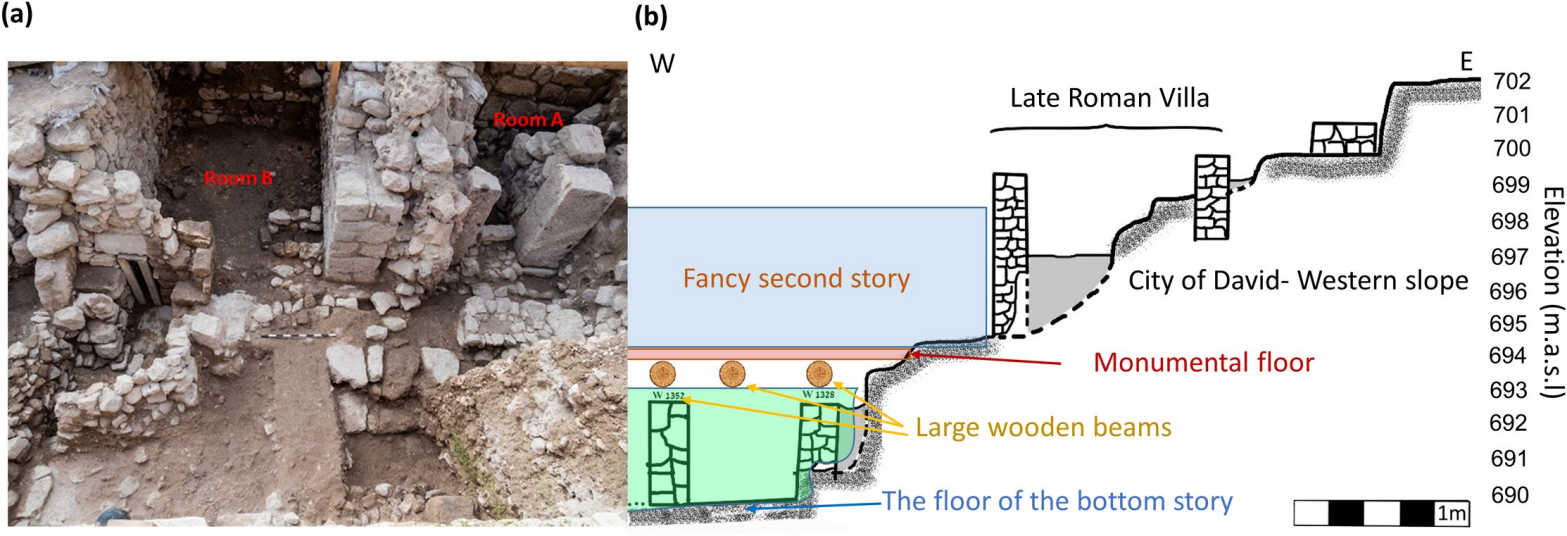
Structure 100 exposed in the Giv’ati Parking Lot excavation. (a) A photo of part of Structure 100, facing south. (b) Suggested reconstruction of Structure 100 on a cross-section of the slope of the site. The light green area marks the eastern part of the bottom story. The second story and its monumental floor are schematically illustrated. Photograph by Assaf Peretz.

Our research was conducted as part of the renewed Giv’ati Parking Lot excavations in Jerusalem (31.7745N/35.2351E) on the western slope of the “City of David” ridge [40]. These excavations exposed a 17X10 m segment of a large structure (Structure 100) that had served as an elite or public building (Fig 1). The bottom story of the structure (Fig 1A) was found filled with a debris layer, up to 2.3 m thick, which included soil and stones, some of which had originated from the second story. Among the debris a substantial amount of ash and charcoal was found, leading the excavators to the conclusion that the structure had been destroyed by an intense conflagration. It is important to note that the structure was built on a steep slope to the west (Fig 1B). Several terraces, each more than five meters wide, were carved into the bedrock, probably to enable this outstanding construction.

Complete pottery vessels of the types common to the Iron Age IIc (the end of the Iron Age, 700–586 BCE [41]) were found within the debris and mainly on the floor of the bottom story of the structure. This pottery assemblage is very similar to that previously discovered in the 586 BCE destruction layers exposed elsewhere in Jerusalem [42–44] and at other sites in the Southern Levant [45]. In particular, a jar handle stamped with a Rosette symbol, characteristic of the royal Judean administrative system, was found within Structure 100. This type of stamped jar handle is typical of the 586 BCE destruction layers exposed in several excavations in Jerusalem and other sites of the former Judean kingdom and has never been found in clear later contexts [46]. A seal and several seal impressions, typical of the end of the Iron Age, were found in the structure as well [47], supporting both its identification as an elite or public building and its dating to the end of the Iron Age. It is important to note that all historical and archaeological data show that there was no major destruction event in Jerusalem for centuries before or after the Babylonian destruction in 586 BCE. These archaeological data and the strong evidence of the destruction of this structure by fire correspond with the biblical description of the conquest and the systematic destruction of Jerusalem by fire in August, 586 BCE. According to this description, this destruction was executed by professional “city destroyers”, under the command of Nebuzaradan, a highly ranked Babylonian official: “And in the fifth month, on the seventh day of the month, which is the nineteenth year of King Nebuchadnezzar King of Babylon, came Nebuzaradan, Captain of the Guard, a servant of the king of Babylon, unto Jerusalem. And he burnt the house of the LORD, and the king's house, and all the houses of Jerusalem, and every great man's house burnt he with fire” (2 Kings, 25, 8–9). Unlike biblical sources regarding earlier periods, the detailed biblical descriptions regarding the end of the Iron Age and specifically the destruction of Jerusalem by the Babylonians in 586 BCE are considered historically reliable by the vast majority of researchers [34, 46, 48]. The above description and other biblical references to this event enable dating of the destruction almost to the day. Besides emphasizing the burning of the Temple and the king’s palace, the biblical author emphasized the destruction of elite houses. The monumentality of the structure discussed above, its total destruction by fire (as confirmed by our results presented below) and its dating to the end of the Iron Age lead to the conclusion that this was one of Jerusalem’s administrative or elite structures destroyed by Nebuzaradan and his forces.

**Fig 2:**
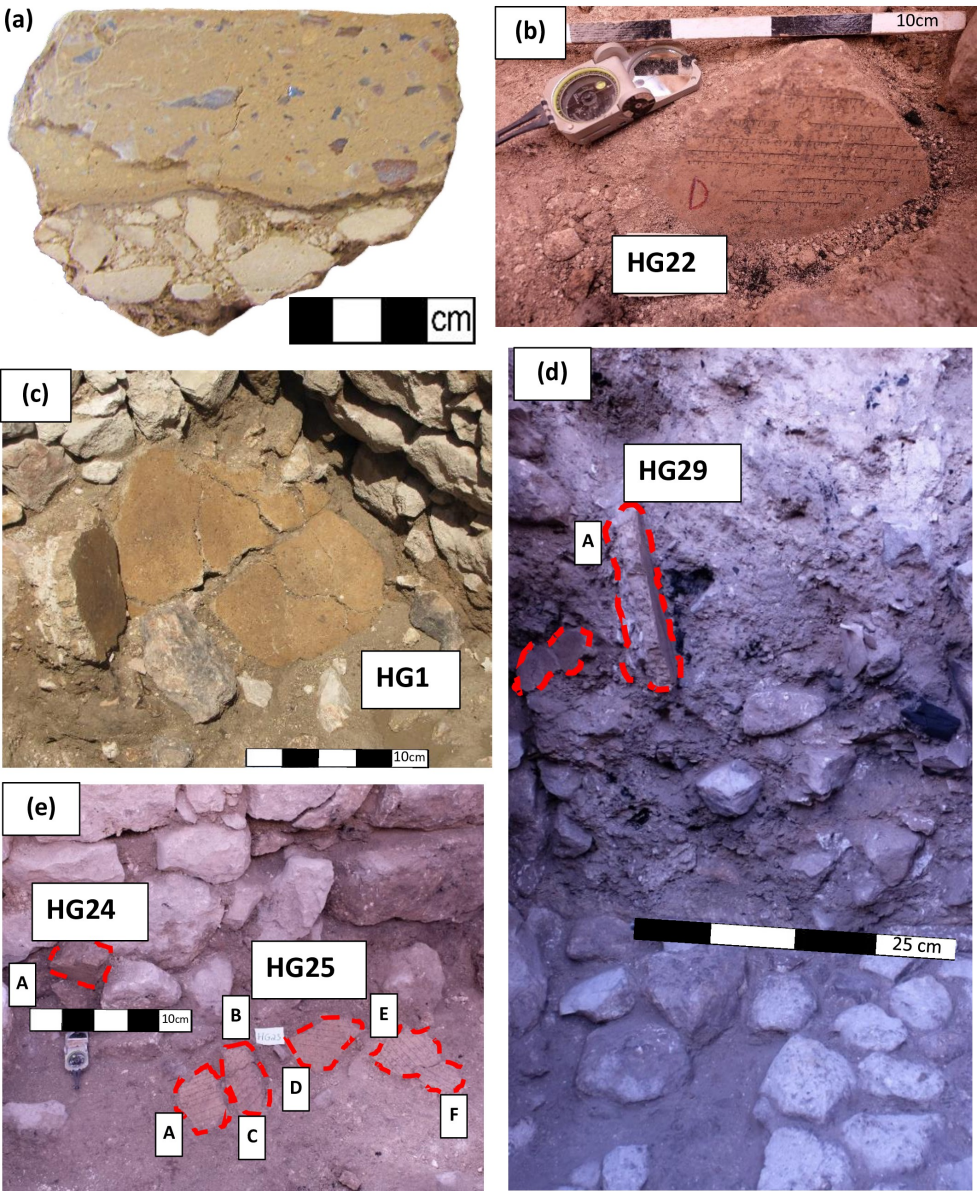
Representative examples of floor segments unearthed in the excavation. (a) A cross-section of a representative floor segment. The bottom layer, only partially visible in this figure, was originally more than 15 cm thick and comprised of small stones. (b) A segment exposed on the floor of the bottom story at a 47° angle from the horizontal plane. (c) A large group of segments exposed articulated (HG1). (d) Two floor segments exposed within the 2.5 m thick debris layer. The segment on the left is facing out of the debris layer whereas the one on the right is facing to the right and slightly up. Large pieces of charcoal and the floor of the bottom story, made of stones, can be seen. (e) Seven floor segments exposed close to the floor of the bottom story. One group of segments (HG25A-F) is lying almost horizontally on the floor of the bottom story, whereas another segment (HG24A) is oriented almost vertically.

Tens of segments of an exquisitely crafted surface were unearthed within the debris of the structure (Fig 2). Fifty-four segments of this surface, most of which are 15 cm thick indicating the thickness of the original surface, were analyzed. The vast majority of the segments consisted of two distinct layers (Fig 2A and S2 Fig). The bottom layer was made of coarse material and pieces of limestone. The upper layer was made of consolidated well-sifted material and contained chunks of calcite, which probably shone when the floor was polished. Its upper face was perfectly flat and smooth. At one location within the structure, four segments of a different floor type were unearthed (HG3A, HG17A, HG23A-B). They were thick and had a flat upper face like the other segments but consisted of one layer only (S3 Fig). These four segments demonstrated magnetic behavior very different from the rest. Their magnetization was approximately 2–3 orders of magnitude weaker than that of the others. All specimens from these segments that had been thermally demagnetized failed criteria. Their susceptibility was under the detection limit of the instrument measuring it (AGICO MFK-1 Kappabridge). For all these reasons, these segments will not be further discussed.

One large group of segments (labeled ‘HG1’) was unearthed articulated in a fallen and crushed position (Fig 2C). All other segments were found separately, at different locations and elevations and in varied orientations throughout the destruction debris, either on the floor of the bottom story or within the debris layer (Fig 2B–2E). Massive charred wooden beams which were found lying on top of a monolithic pier and among the debris, including below HG1, raised the possibility that the surface had originally been supported by them and served as the floor of the second story of the monumental building. No comparable floor from the Iron Age has ever been found in Jerusalem or other sites in the southern Levant. There were no definitive signs of fire observed directly on the floor. However, the massive burnt wooden beams and other signs of burnt material led us to hypothesize that the failure of these beams during the conflagration resulted in the collapse of the floor. Due to the large terraces carved into the bedrock of the steep slope we assume that the main entrance to the building was from the east, directly into the second story (Fig 1B; note that the walls of the Roman Villa were constructed after Structure 100 had gone out of use). This explains, to our understanding, the great investment in the construction of the floor of the second story.

## Materials and methods

The strategy of the paleomagnetic analyses was as follows. First, we carried out paleomagnetic demagnetization experiments using one of two methods described below in order to isolate different components of the paleomagnetic vector. We then calculated the averaged paleomagnetic direction of each segment in an attempt to find out which of the floor segments had cooled in-situ and which had moved significantly after cooling. Segments that yielded directions far away from the others and more than 40° away from the average direction (GAD) of the normal field in Jerusalem (declination = 0°, inclination = 51°) were considered to have shifted significantly after cooling and were therefore screened out. We then calculated the mean direction of the remaining floor segments. To estimate the field intensity (paleointensity) we carried out absolute paleointensity experiments. These analyses provided us with a full vector representation of the ancient field at the time of the destruction. In addition, thermomagnetic curves were measured in an effort to characterize the response of the floor material to heat, add constraints to the heating temperatures and estimate the mineralogy of the ferromagnetic carriers. Hysteresis and first order reversal curves (FORC) were measured in order to further characterize the nature of the magnetic carriers. In the following paragraphs we will describe in detail the experiments mentioned above.

We sampled the floor segments by measuring the strike and the dip of the segments’ flat surfaces at several points using a Brunton compass and marking horizontal lines parallel to the strike (Fig 2B). A sun compass was used in the field in order to correct the possible influence of local magnetic anomalies on the magnetic measurements. No method, such as the common use of Plaster of Paris, was necessary in order to create flat surfaces since the segments were perfectly flat to begin with. From each segment we cut several (6–10) square shaped specimens, with one edge of the square cut parallel to the strike. Specimens for alternating field (AF) demagnetization were glued in non-magnetic paleomagnetic sampling boxes, 1.5x2x2 cm in size. Specimens for thermal demagnetization were glued in square alumina crucibles, 1.9x2.1x2.1 cm in size.

Paleomagnetic experiments were carried out at the magnetically shielded paleomagnetic laboratory at the Institute of Earth Sciences, the Hebrew University of Jerusalem, using a 2G enterprises RAPID super conducting rock magnetometer (SRM) system with in-line 2-axis AF demagnetizer and paleomagnetic ovens modified from ASC-TD-48. Magnetic susceptibility was measured using an AGICO MFK-1 Kappabridge with a CS4 furnace. Magnetic hysteresis and FORC measurements were carried out using a Lakeshore 8604 vibrating sample magnetometer (VSM) and analyzed using FORCinel program [49].

AF demagnetization was carried out at progressively elevated peak fields in 4mT steps up to 20mT, 5mT steps up to 40mT, 10mT steps up to 70mT and 15mT steps up to 100 mT. Thermal demagnetization was carried out at progressively elevated temperatures, in 50°C steps from 100°C to 200°C, 40°C steps up to 400°C, 30°C steps up to 490°C and two high temperature steps at 540°C and 600°C. Paleointensity experiments were carried out following the Thellier-IZZI protocol [50, 51] at progressively elevated temperatures, in 50°C steps from 100°C to 200°C, 20°C steps up to 320°C and a high temperature step at 470°C, with pTRM checks [52] at every second temperature step. Anisotropy experiments included eight steps at 470°C: a demagnetization step, six TRM acquisition steps at six orthogonal orientations (+x, -x, +y, -y, +z, -z) and an alteration check step. Cooling rate experiments included three steps at 470°C: fast rate (fan cooled, averaged rate of 27°C per minute), slow rate (averaged rate of 50°C per hour) and an alteration check at the fast rate.

Paleomagnetic data were analyzed using the PmagPy software package [53]. Demagnetization data were analyzed using the Demag GUI program, where best-fit paleomagnetic directions were calculated using principal component analysis [54]. Paleomagnetic means were calculated and the statistical parameters were determined using Fisher statistics [55]. All field orientations were corrected using sun compass measurements. Paleointensity data were analyzed using the Thellier GUI program [56] following the automatic interpretation approach [32, 57]. Here, we used the same acceptance criteria as in Shaar et al. [32] and Ben-Yosef et al. [20] (S5 Table). All the paleomagnetic and the paleointensity data, as well as the interpretation presented here, were uploaded to the MagIC database (https://www2.earthref.org/MagIC/16802).

Thermomagnetic curves were measured for seven floor segments in repeated cycles at progressively elevated peak temperatures from 100°C up to 700°C, in 100°C steps, in an oxidized environment in order to approximate conditions of the fire during the destruction.

Magnetic hysteresis and back-field IRM curves were measured for 18 specimens, one specimen from each floor segment that was sampled for paleointensity. As all specimens yielded nearly identical hysteresis parameters, a FORC distribution was measured for one of these specimens using 800 loops.

All necessary permits were obtained for the described study, which complied with all relevant regulations. The archaeological samples were excavated under license number G-11/18 by the Israel Antiquity Authority. All samples are stored in the archaeomagnetic laboratory at Tel Aviv University and are available for study: HG1, HG5, HG6, HG12, HG13, HG14, HG18, HG20, HG21, HG22, HG24, HG25, HG27, HG28, HG29 (for more information see: S1, S2 and S3 Tables). Specimens from all these samples that were measured in the different experiments are stored in the paleomagnetic laboratory at the Institute of Earth Sciences, the Hebrew University of Jerusalem and are available for study as well.

## Results

### Demagnetization experiments

**Fig 3:**
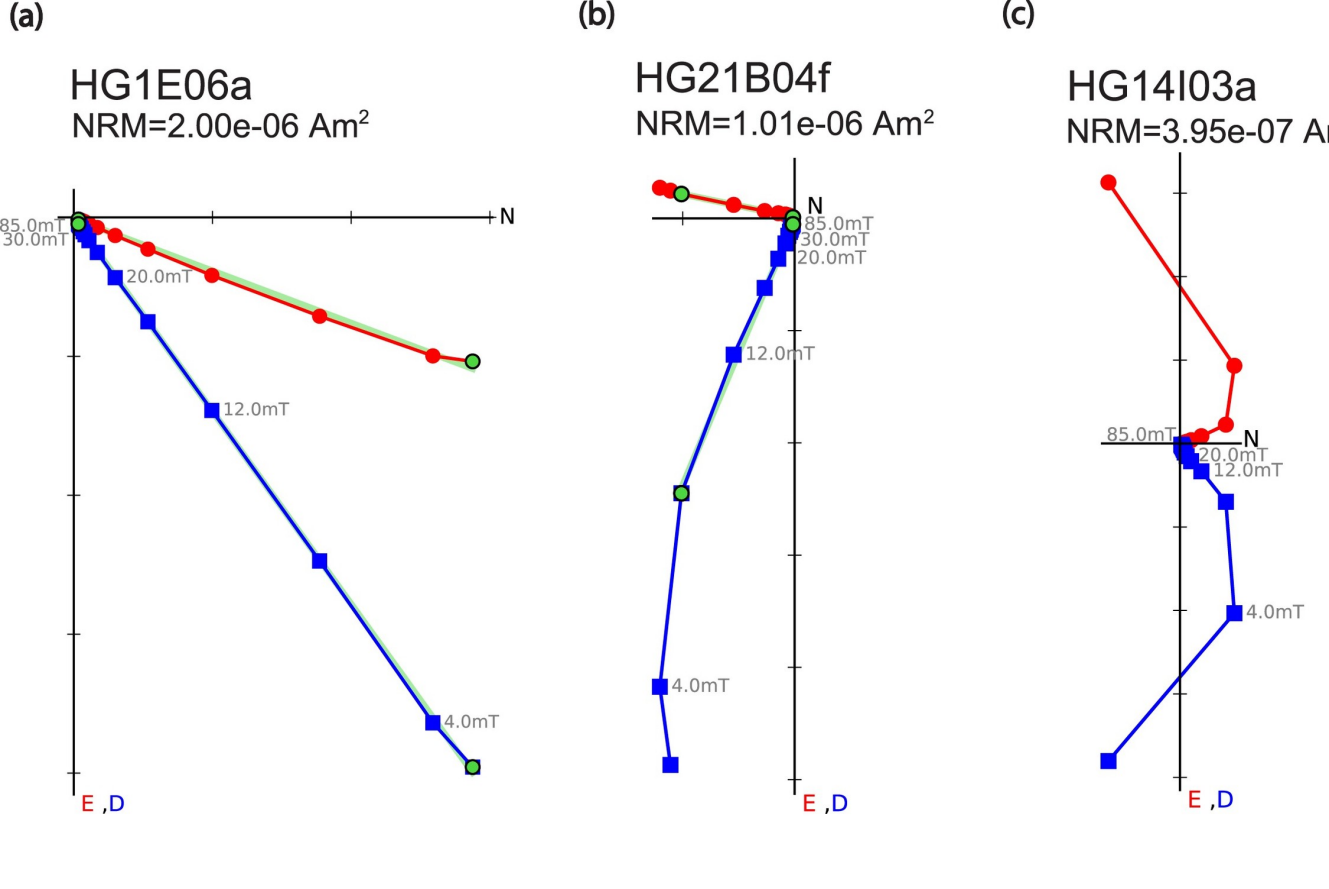
Representative results of AF demagnetization experiments displayed as Zijderveld [58] end-point orthogonal diagrams. Red circles (blue squares) are projections on the north-east (north-down) plane. **(a)** Ideal behavior meeting selection criteria with straight lines converging to the origin indicating a stable magnetization with northeastern declination and positive inclination. **(b)** A primary magnetization with southern declination and steep (~70°) inclination. **(c)** Specimen rejected based on curvature of the Zijderveld diagram.

In total, 397 specimens from 54 floor segments underwent a stepwise demagnetization experiment: 375 using AF and 22 using thermal techniques. S4 Fig shows the coercivity spectra of all specimens analyzed, indicating that the median destructive field (MDF) is between 4 and 12 mT and that 90% of the natural remanent magnetization (NRM) is removed after 30 mT. The vast majority of specimens yielded straight Zijderveld diagrams [58] with a univectorial component converging to the origin (Fig 3A). Other behaviors (e.g. Fig 3C) were screened out based on the maximum angle of deviation (MAD) [54] and the deviation angle (DANG) [50] statistics, where specimens with values exceeding five were rejected. Out of 397 specimens, 341 met these criteria. Most specimens yielded a vector pointing roughly to the north and down, around the average direction of the normal geomagnetic field in Israel (e.g. Fig 3A). However, other specimens yielded directions that are completely different from this direction (e.g. Fig 3B).

**Fig 4:**
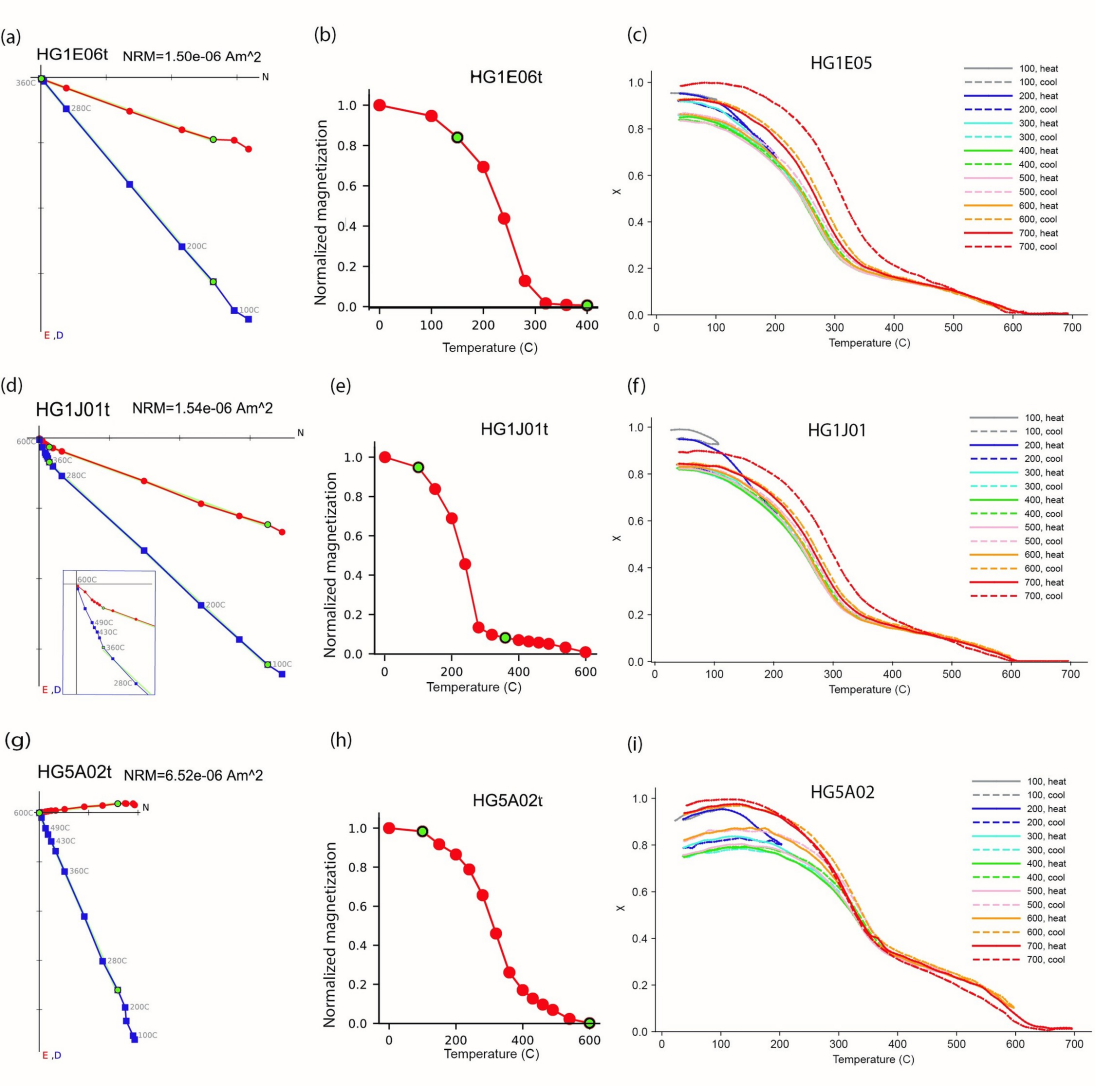
Representative results of thermal demagnetization and thermomagnetic curves. Each row shows results from the same floor segment. **(a)**, **(d)** and **(g)** display Zijderveld [58] plots, where symbols and colors are as in Fig 3. Inset in (d) displays a zoom-in view of the 280–600°C steps. **(b)**, **(e)** and **(h)** display the normalized magnetization versus temperature step. **(c)**, **(f)** and **(i)** display the magnetic susceptibility measured in repeated heating cycles at progressively elevated peak temperatures. (a-c) show the behavior of most of the specimens with magnetization removed at 320°C. (d-i) show two examples representing specimens which were completely demagnetized only at 600°C. All thermomagnetic curves show one significant drop between 300–350°C and a smaller drop around 600°C.

Nineteen of the 22 thermally demagnetized specimens from 19 floor segments showed a similar behavior of a univectorial magnetic component converging to the origin (Fig 4A and 4G) and a very good agreement with the AF results (e.g. Fig 3A versus Fig 4A). Only two specimens failed the MAD and DANG criteria. One specimen yielded a magnetic vector comprised of two distinct components, one of which was erased during lower temperature steps (Fig 4D). S5 Fig shows the blocking temperature spectra of all specimens, indicating that the mean destructive temperature (MDT) of most specimens is between 140° to 240°C. A near complete removal of the NRM at 250°-320°C was measured in most cases (Fig 4A and 4B, S5A Fig), with three exceptions. In two specimens (e.g. Fig 4D and 4E, S5A Fig) most of the magnetization was removed at 280°C but it was entirely removed only at 600°C. In one other specimen (Fig 4G and 4H, S5A Fig) the magnetization was gradually removed until 600°C.

### Thermomagnetic curves

Seven specimens from different floor segments showed similar thermomagnetic curves, indicating similar bulk magnetic mineralogy. The curves are nearly reversible, demonstrating little alteration and thus stability of magnetic minerals up to 600°C. Three representative curves, alongside the matching thermal demagnetization results from the same segments, are shown in Fig 4C, 4F and 4I. The main drop in the susceptibility between 300°C to 350°C indicates that the Curie temperature (T_c_) of the main magnetic component is around these temperatures. Another smaller drop is observed between 550°C to 580°C, indicating that magnetite in small quantities is also present.

### Paleomagnetic directions

**Fig 5:**
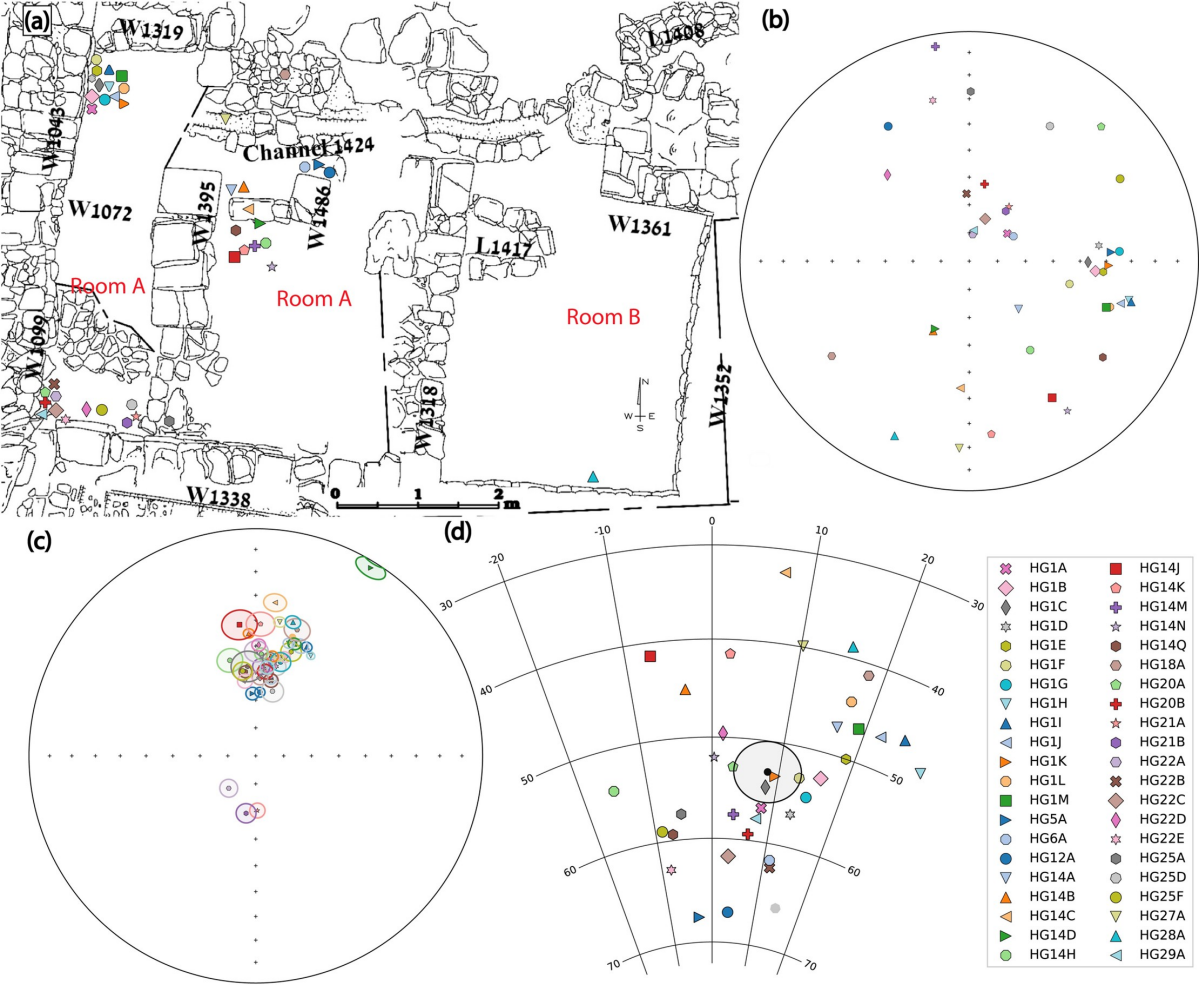
Locations, orientations and mean archaeomagnetic directions of floor segments. **(a)** Location map of the floor segments that met acceptance criteria. **(b)** The orientation (dip and dip direction) of the floor segments’ flat surface in (a) plotted on an equal area projection. **(c)** Mean directions of the segments in (a)-(b) with their α_95_ cones plotted on an equal area projection. **(d)** Mean directions from (c) excluding directions failing the GAD criterion (see text for details). The average direction and the α_95_ cone of these segments are plotted in black, representing the mean paleomagnetic direction of the entire burnt floor.

We averaged the paleomagnetic directions of every floor segment separately and calculated the statistical parameters assuming a Fisherian distribution [55]. As we wished to isolate only floor segments that reliably recorded the ancient field, we applied the following acceptance criteria: number of specimens n ≥ 4; precision parameter *k* ≥ 50; 95% confidence cone α_95_ ≤ 6°. Forty-two out of 54 floor segments met the criteria (S1 and S2 Tables). These segments show relatively tight clusters with *k* exceeding 100 and typical α_95_ values below 3°. S3 Table lists the results of the 12 floor segments that did not meet criteria. The 42 segments that met criteria were unearthed in different locations as shown in Fig 5A. While the segments are inclined with respect to the horizontal plane at varying angles ranging from 6° to 80° in a random fashion (Fig 5B, S1 and S2 Tables), their paleomagnetic means are clearly not random, as seen in Fig 5C. Out of these 42 segments, 38 adhere to the expected direction of the field in Israel, yielding northerly declinations and inclinations ranging between 33° and 66°, scattered around the geocentric axial dipole (GAD) inclination in Jerusalem (51°).

In order to estimate the direction of the ancient magnetic field, we averaged the directions of the 38 segments that yielded directions clustered around the average GAD direction in Israel and calculated the statistical parameters assuming a Fisherian distribution (Fig 5D). This was done under the assumption that these segments, unearthed in different locations and orientations, experienced slight and random movement since they had cooled down. The mean direction and its confidence cone (declination = 8.3°, inclination = 53.1°, n = 38, *k* = 59, α_95_ = 3.0°) are shown in Fig 5D.

**Fig 6:**
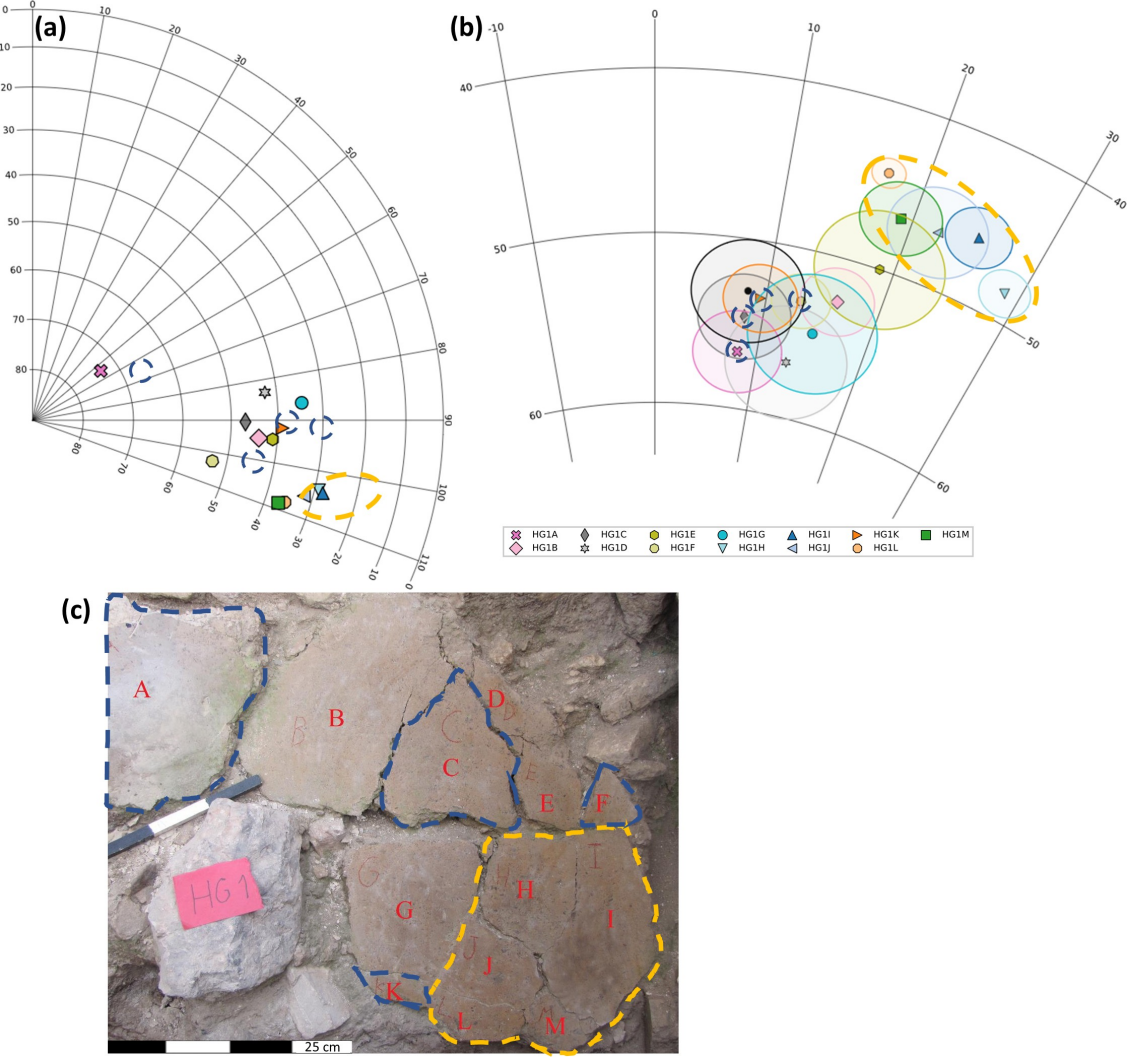
Paleomagnetic direction of HG1 –a cluster with 13 tilted floor segments. Segments H, I, J, L and M are marked by a yellow dashed line. Segments A, C, F and K are marked by a blue dashed line. **(a)** The orientation (dip, dip direction) of the segments’ flat surface. **(b)** The mean paleomagnetic directions of the floor segments with their corresponding α_95_ confidence cones plotted on an equal area projection. The mean direction of all 38 segments (Fig 5D) and the corresponding α_95_ confidence cone are shown in black. **(c)** A photo of HG1 where letters denote the different segments.

HG1, the only large group of segments that was unearthed articulated, enabled us to examine in detail the collapse and cooling processes that occurred. By comparing the field orientations, locations and direction results of the 13 segments of HG1 (Fig 6), we were able to estimate which segments had cooled in situ and which had moved significantly after cooling. Five segments of HG1, namely H, I, J, L and M (marked by a yellow dashed line in Fig 6), were all originally connected to each other (Fig 6C) and were unearthed at similar tilt angles (Fig 6A and S1 Table). The difference between the tilt angles of this group and most other segments of HG1 is in accordance with the difference in magnetic direction calculated from them (Fig 6A and 6B). Thus, it seems very likely that these segments moved as a group away from the rest after they had cooled down, resulting in the diversion of their magnetic vectors. On the other hand, segments A, C, F and K (marked by a blue dashed line in Fig 6), yielded paleomagnetic directions statistically equivalent to the calculated average of all 38 segments. These four segments were unearthed at some distance from each other (Fig 6C) and at different tilt angles (Fig 6A and S1 Table). It is therefore unlikely that after these segments had cooled down, they all moved in a manner that on the one hand kept the magnetic vectors clustered together but on the other hand tilted these segments in different orientations.

### Absolute paleointensity

**Fig 7:**
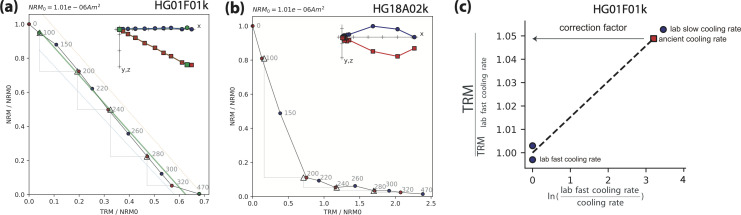
Representative results of the paleointensity experiments. **(a)** A representative Arai plot of a specimen meeting acceptance criteria. Red circles, blue circles and triangles represent ZI steps, IZ steps and pTRM checks, respectively. Blue and red squares in the Zijderveld plot (inset) represent x–y and x–z projections of the NRMs in the specimen coordinate system, respectively, where x-axis is rotated to the direction of the NRM. Best-fit lines and temperature bounds are marked in green. **(b)** Specimen not meeting criteria. **(c)** A representative result of a cooling rate correction experiment following Halgedahl et al. [60]. Graphs **(a)** and **(c)** both represent results of experiments carried out on the same specimen.

For paleointensity analysis we analyzed 36 specimens prepared from 18 different floor segments among the 38 segments that had yielded directions clustered around the average direction in Israel (Fig 5C and 5D and see above). Fig 7 shows representative results of the paleointensity experiments. Out of 36 specimens, 30 met our acceptance criteria (S5 Table) [20, 32, 57], which is a relatively high success rate. The specimens that met criteria demonstrated a nearly ideal behavior, characterized by a linear Arai plot with pTRM checks (triangles in Fig 7A) overlapping the infield data points (red circles) and a straight Zijderveld plot (inset in Fig 7A) converging to the origin. All specimens were corrected for anisotropy and cooling rate effects. For cooling corrections, we assumed that the temperature dropped from 300°C to 100°C in four hours. Our use of this rate, which was estimated from the experimental results of Kreimerman and Shahack-Gross [59], resulted in a cooling rate correction ranging between 4–7% (S6 Fig). Had we adopted a much slower cooling rate of eight hours from 300°C to 100°C the change in the cooling rate correction factor would have been negligible. The anisotropy correction was typically less than 5% (S7 Fig).

The paleointensity result of one specimen (HG18A02i) was discarded as an outlier since it yielded a result more than 19% lower than the mean of the other 29 specimens whereas the 95% confidence interval of the other 29 specimens is less than 2.2%. The mean paleointensity of the 29 specimens was calculated using two calculation methods. In the first method, the Thellier GUI program algorithm collects all the acceptable interpretations from each specimen and chooses a set of discrete interpretations that minimizes the standard deviation of the mean [56]. In the second method, the range of the acceptable interpretations of each specimen (S4 Table) defines a paleointensity interval. This interval is used as a uniform distribution function from which a random value is drawn in a bootstrap procedure [61]. Both calculations yielded similar results, where the arithmetic paleointensity mean is 78.2 ± 1.7 μT and the bootstrap paleointensity mean is 78.1 μT with 95% confidence interval between 77.4 μT and 78.7 μT (S8 Fig). These values correspond to a virtual axial dipole moment (VADM) of 149.3 ± 3.2 ZAm^2^ and 149.2 (147.8–150.4) ZAm^2^ for the arithmetic mean and bootstrap mean, respectively. The arithmetic mean will be used from here on.

### Hysteresis and FORC measurements

All 18 hysteresis loops yielded very similar results (S9 Fig), clustered together on the Day plot [62, 63] (panel (a) in S9 Fig), which implies homogeneousness of the magnetic carriers in these specimens. The hysteresis parameters fall outside the titanomagnetite single domain (SD), pseudo single domain (PSD) and multi domain (MD) fields of the Day plot [63] with squareness (Mr / Ms) values around 0.18, coercivity (Bc) between 4 to 5 mT, coercivity of remanence (Bcr) between 5 to 8 mT and Bcr / Bc around 1.4. However, the FORC diagram (panel (d) in S9 Fig), which provides detailed information on the distribution of coercivities and interaction fields, shows a very clear single domain behavior with a characteristic narrow ridge along the coercivity (Bc) axis spanning up to 30 mT. The FORC also shows a weak multi-domain (MD) signal, superimposed on the SD distribution, with a large vertical spread near Bc = 0. This demonstrates the high quality of the floor material as a magnetic recorder and further validates the paleointensity results presented above.

## Discussion

The rare combination of reliable historical data and related burnt archaeological materials enabled us to closely study the formative event of Jerusalem’s destruction by archaeomagnetic methods. Archaeomagnetic tools allowed us to reconstruct the site formation processes and show that many segments of a thick surface found inside a recently excavated debris layer had originally been the floor of a monumental structure. The demagnetization experiments showed that most of the floor segments, found in an extensive area of the excavation (Fig 5A) and at various tilt angles ranging from nearly horizontal to nearly vertical, had cooled in a position similar to that in which they were unearthed, preserving the magnetic field direction. However, four segments yielded directions that deviate significantly from the average direction of the field. These segments probably moved from the orientation in which they had cooled down. It is possible that they had cooled down in their original position and then collapsed or that they had collapsed, cooled down and then underwent further movement. Such movement, due to post depositional processes, is expected to occur during extended periods, in particular in the case of debris layers that include organic matter and air cavities. Our results provide insights regarding the original architecture of the monumental building. They reinforce the understanding that the floor had originally been part of the second story of the structure and that it collapsed during the conflagration, due to the failure of the massive wooden beams supporting it (Fig 1B).

Some specimens yielded a magnetic vector comprised of two components (e.g. Figs 3B and 4D) or even more (Fig 3C). It seems very likely that these specimens moved during their cooling process. Therefore, the field direction recorded at the lower temperatures (360°C and lower in the case presented in Fig 4D) was probably recorded after the movement and thus better represents the direction of the field during the destruction. Furthermore, we assume that at least some of the floor segments that did not meet criteria moved during the cooling process. On the specimen level, such movement can result in multiple components of the magnetic vector, high MAD and DANG and rejection of the specimen. On the segment level, such movement can result in scattered direction results within a certain floor segment due to heterogeneous temperature distribution while cooling. Such differences in temperature could have occurred due to differences in the air flow or in distance from the heat sources. Rejection of some specimens or scattered direction results could both result in the rejection of the segment.

The experimental results show that most of the magnetic information recorded in the floor segments was acquired by heat, namely TRM. TRM can probably explain even the multiple components of magnetization demonstrated by some specimens. The thermal demagnetizations show that the magnetization was entirely removed after 280–320°C for most floor segments. This provides a minimum constraint to the heating temperature of these segments during the conflagration. However, for three floor segments that were entirely thermally demagnetized only at 600°C, the minimum heating temperature during the destruction was ca. 600°C. The close vicinity between some of the segments with different minimum temperatures (e.g. Fig 4A–4C versus Fig 4D–4F) suggests that the segments that were demagnetized at lower temperatures might have been heated to ca. 600°C as well. In that case, the different results in the thermal demagnetization are the result of differences in the mineralogy of the different segments. The thermomagnetic curves provide additional insights, since all specimens demonstrated stability of the magnetic minerals while being heated in the lab to 600°C. This stability might be the result of the heating during the conflagration to ca. 600°C [64]. We can conclude that the temperature of the conflagration, at least in some areas, reached ca. 600°C.

The investigation of site-formation processes presented above is valuable not only for the archaeological research of the site, but also for strengthening the validity and the dating of the archaeomagnetic results. The paleomagnetic direction data, combined with archaeological observations and the insights regarding the temperature of the fire, enabled us to strongly connect the collapse of this structure with the conflagration event. Our results show that the acquisition of the magnetic information in the floor segments occurred during the destruction of the structure by fire. From this, in conjunction with the historically-based tight dating, we can conclude that we managed to pinpoint our paleomagnetic results on the time axis to less than a year, which is rare for such an early period.

**Fig 8:**
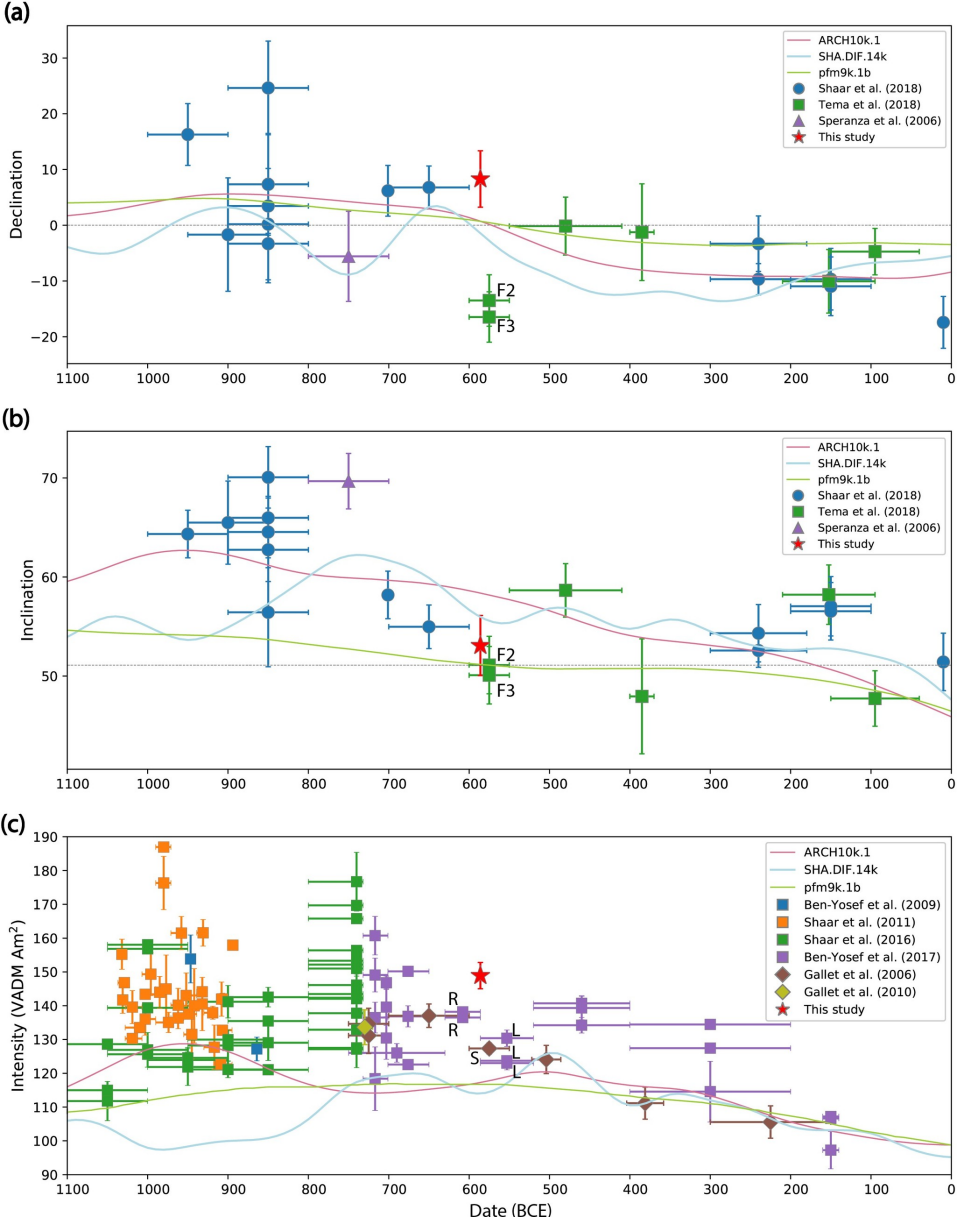
Archaeomagnetic direction and intensity data in the Levant (Israel, Syria, Jordan, Cyprus). The average results from the 586 BCE destruction layer in Jerusalem are represented by red stars and error bars **(a)** Declination. **(b)** Inclination. All directions are relocated to Jerusalem. The dashed grey lines represent the direction of a geocentric axial dipole (GAD) field in Jerusalem. The results of two chambers of a twin furnace from the Agia Varvara-Almyras archaeological site are marked “F2” and “F3”. (**c)** Paleointensity given as the corresponding virtual axial dipole moment (VADM). The colored curves represent three different global geomagnetic models [1–3]. The results of two types of Judean stamped jar handles are marked: Rosette jar handles with the letter “R” and Lion jar handles with the letter “L”. The result from Sheikh Hamad site is marked with the letter “S” (see text for details).

In Fig 8 we present the direction and intensity results from this research and previously published archaeomagnetic data from the nearby regions (Israel [20, 21, 30, 32], Jordan [31], Cyprus [65] and Syria [66–69]) between 1200 BCE and the beginning of the Common Era.

The new well-dated archaeomagnetic data might help constrain the duration of the Levantine Iron Age Anomaly (LIAA)–a high field anomaly, which spanned from the 10^th^ century BCE until at least the 5^th^ century BCE in the Near East and Europe [5, 21, 35–37]. This anomaly is characterized by generally high averaged field values (130–160 ZAm^2^), with short episodes of higher field values and geomagnetic spikes, fast secular variation rates and large angular deviations from the GAD field. In the Levant, the termination of the LIAA was constrained to the 7^th^ century BCE by moderate field values measured from Judean stamped pottery [20] and pottery from Syria [66, 70]. The new high field data from 586 BCE, with field value of 149 ZAm^2^, suggest instability of the field during the 6^th^ century BCE. This could be explained as part of the LIAA, stretching its duration at least until 586 BCE. However, unlike the period between the 10^th^ and the 8^th^ centuries that was characterized by large angular deviations from the GAD field [21, 32], the direction of the field in 586 BCE deviated only a few degrees from the GAD direction. Finally, we note that the paleointensity is much higher than predicted by global models [1–3], the declination is somewhat higher than predicted and the inclination is in agreement with the models.

The direction and intensity data, well dated to the 586 BCE destruction of Jerusalem which marks the end of the Iron Age in the Levant, can serve as chronological markers for archaeomagnetic dating. This is of special importance for archaeological and historical considerations. The paleointensity results presented here were compared to previous paleointensity results from the Levant, including Judean stamped jar handles (purple symbols in Fig 8C) [20]. The 586 BCE new results show considerably higher field values than those measured from the Rosette type (marked “R” in Fig 8C) that were produced before the 586 BCE destruction and from the Lion type (marked “L” in Fig 8C) produced after it. It is widely accepted that the Rosette stamp system went out of use in 586 BCE since complete storage jars with handles stamped with a Rosette stamp seal were found in clear 586 destruction contexts, including one within the structure discussed here, and none were found in clear later contexts. More precise dating suggestions were based mainly on historical interpretations. Some scholars interpreted the Rosette handles as part of the preparation of the Judean Kingdom for the Babylonian campaign and thus dated the manufacture of all of them to the several years preceding 586 BCE [45]. Our intensity results compared to those of two different Rosette handles [20] show a VADM difference of more than 10 ZAm^2^. These results rule out the possibility that all the Rosette handles were manufactured so close to the 586 BCE destruction and support an earlier date for the beginning of their manufacture. The production could have commenced during the last decade of the 7^th^ century BCE [71] but probably began a few decades earlier. Our results support the historical understanding that the Rosette handles were not manufactured as part of the preparations of the Judean kingdom for the Babylonian campaign. For historical considerations, it seems likely that the Rosette handles were manufactured over a period of decades, like other royal Judean stamped jars, as part of the ongoing administrative system of the Judean kingdom [46]. These results can help determine what the purpose of this system was and constrain the dating of other sites in which Rosette handles have been unearthed. For similar reasons, the three Lion stamped jar handles [72] that were measured for paleointensity in previous research [20] had probably been manufactured at least a few decades after 586 BCE.

Our intensity results are ca. 20 ZAm^2^ higher than those measured from a collection of pottery sherds dated to 600–550 BCE (a brown symbol marked “S” in Fig 8C), from room CW in the “Red House” at the Sheikh Hamad archaeological site in Syria [66, 68, 73, 74]. The discrepancy can be due to a rapid change in the field’s intensity, similar to the changes during the “Iron Age Spikes” [30]. Another possible explanation could be the inherent difficulty in dating the acquisition of the magnetic signal in pottery sherds.

Our declination result is ca. 23° higher than that measured from two chambers of a twin furnace, labeled “Furnace 2” and “Furnace 3” (green symbols in Fig 8A and 8B marked “F2” and “F3” respectively) sampled in the Agia Varvara-Almyras archaeological site in Cyprus. This twin furnace was dated using three radiocarbon measurements of charcoal unearthed within it. More precise dating to 600–550 BCE was then obtained by narrowing the radiocarbon intervals using stratigraphic position, diagnostic waste layers and pottery typology. The discrepancy between these results and the 586 BCE result presented here can be explained by a fast change in declination during this period. The discrepancy can also be explained by the inherent difficulty in precise dating based on radiocarbon in this period and/or the difficulty in obtaining accurate directions from collapsed materials. The scattered direction results of the floor segments in the current study, with Fisher precision parameter (k) = 59, demonstrate a disadvantage of sampling collapsed material for paleodirection. Future research of other sites destroyed during the 586 BCE Babylonian campaign will help corroborate the results presented here.

As demonstrated above, the new 586 BCE chronological marker can help constrain the chronology of previous and future archaeomagnetic data from the same period and help validate suggested links between the Babylonian 586 BCE campaign and destruction layers in other sites. In this period, which is in the middle of the Hallstatt Plateau [33], dating using radiocarbon is very limited in its precision, due to the flat nature of the calibration curve. Therefore, archaeomagnetic dating using the new results as a chronological benchmark has a great advantage as a dating tool in this period. It can be used both for archaeomagnetic dating of other destruction layers and other finds that have not been dated historically and for the research of the enigmatic behavior of the ancient magnetic field in this period and region.

## Supporting information

S1 Fig Synthetic radiocarbon dating for 586 BCE. A theoretical date of 2485±25 (BP) which corresponds to 586 calBC (by reverse calibration) and its calibrated results.
(TIF)

S2 Fig A cross-section of a representative floor segment. Only the top layer is fully visible. The bottom layer, only partially visible in this figure, was originally more than 15 cm thick and comprised small stones. Photograph by Sasha Flit.
(JPG)

S3 Fig A cross-section of one of the four “chalky” segments. These segments consist of a single layer. Photograph by Sasha Flit.
(JPG)

S4 Fig Coercivity spectra from all AF demagnetization experiments. **(a)** The normalized vector difference sum (VDS) versus the AF demagnetization step. The different colors represent different ranges of the median destructive field (MDF), which is the peak AF field value (mT) needed to reduce the VDS to 50% of its initial value. **(b)** A histogram of the MDF of all specimens from AF demagnetization.
(EPS)

S5 Fig Blocking temperature spectra from all thermal demagnetization experiments. **(a)** The normalized vector difference sum (VDS) versus demagnetization steps. The different colors represent different ranges of the median destructive temperature” (MDT), which is the temperature (C°) needed to reduce the VDS to 50% of its initial value. **(b)** A histogram of the MDT of all specimens from thermal demagnetization.
(EPS)

S6 Fig A histogram of the cooling rate correction factors. (JPG)

S7 Fig A histogram of the anisotropy correction factors. (JPG)

S8 Fig Paleointensity bootstrap results. The dots show the paleointensity interpretations for each specimen that passed criteria. Vertical lines show the range of B_min_ and B_max_ from Table S4. The horizontal green line shows the bootstrap mean and the 95% confidence interval.
(EPS)

S9 Fig Hysteresis and FORC results. **(a)** Day plot [62] of all 18 specimens measured. The titanomagnetite single domain (SD), pseudo single domain (PSD) and multi domain (MD) areas are marked following Dunlop (2002). **(b)** Hysteresis loop of HG01A11a, characteristic of all specimens measured. The saturation magnetization (marked Ms), the saturation remanence (the y-intercept, marked Mr) and the coercivity (x intercept or Bc) are presented. **(c)** Back-field DCD plot of HG01A11a, same specimen as in (b), characteristic of all specimens measured. The coercivity of remanence (marked Bcr) is presented. **(d)** A first order reversal curve (FORC) diagram of HG01A11a, same specimen as in (b) and (c) calculated from 800 equally spaced loops measured with saturation field of 1T and averaging time of 100mT; SF = 4.
(EPS)

S1 Table Paleomagnetic directions of segments meeting all criteria. (PDF)

S2 Table Paleomagnetic directions of fragments meeting criteria, but with directions not clustered around the GAD field. (PDF)

S3 Table Paleomagnetic directions of floor segments that failed criteria. (PDF)

S4 Table Specimen paleointensity results. (PDF)

S5 Table Paleointensity acceptance criteria. (PDF)
