# Supplementary figures and images for: The Earth’s magnetic field in Jerusalem during the Babylonian destruction: A unique reference for field behavior and an anchor for archaeomagnetic dating
Source: PLoS One. 2020 Aug 7;15(8):e0237029. doi: 10.1371/journal.pone.0237029 (PMC7413505; doi:10.1371/journal.pone.0237029)

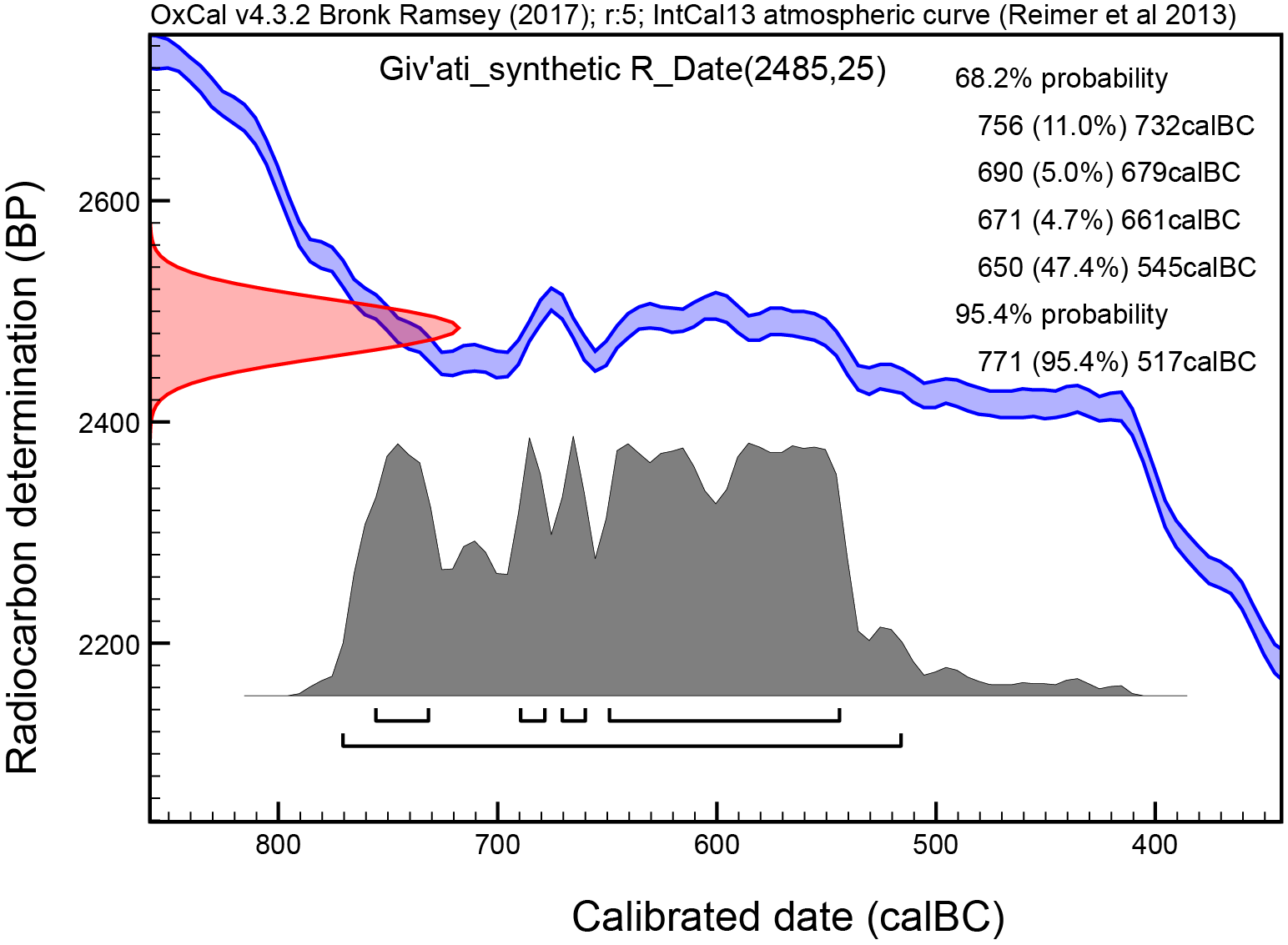

Supplement: S1 Fig — A theoretical date of 2485±25 (BP) which corresponds to 586 calBC (by reverse calibration) and its calibrated results. (TIF) [file pone.0237029.s001.tif]

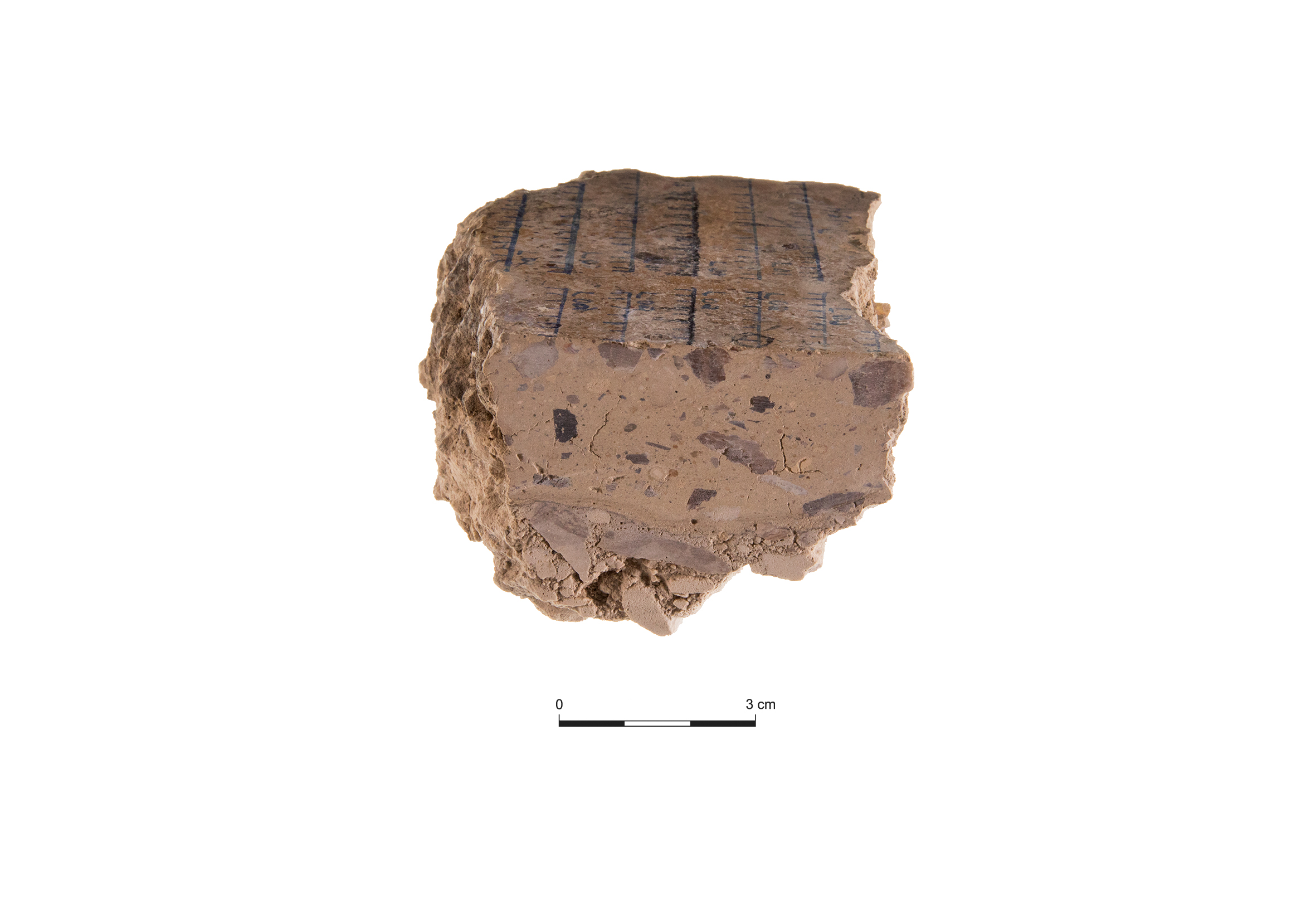

Supplement: S2 Fig — Only the top layer is fully visible. The bottom layer, only partially visible in this figure, was originally more than 15 cm thick and comprised small stones. Photograph by Sasha Flit. (JPG) [file pone.0237029.s002.jpg]

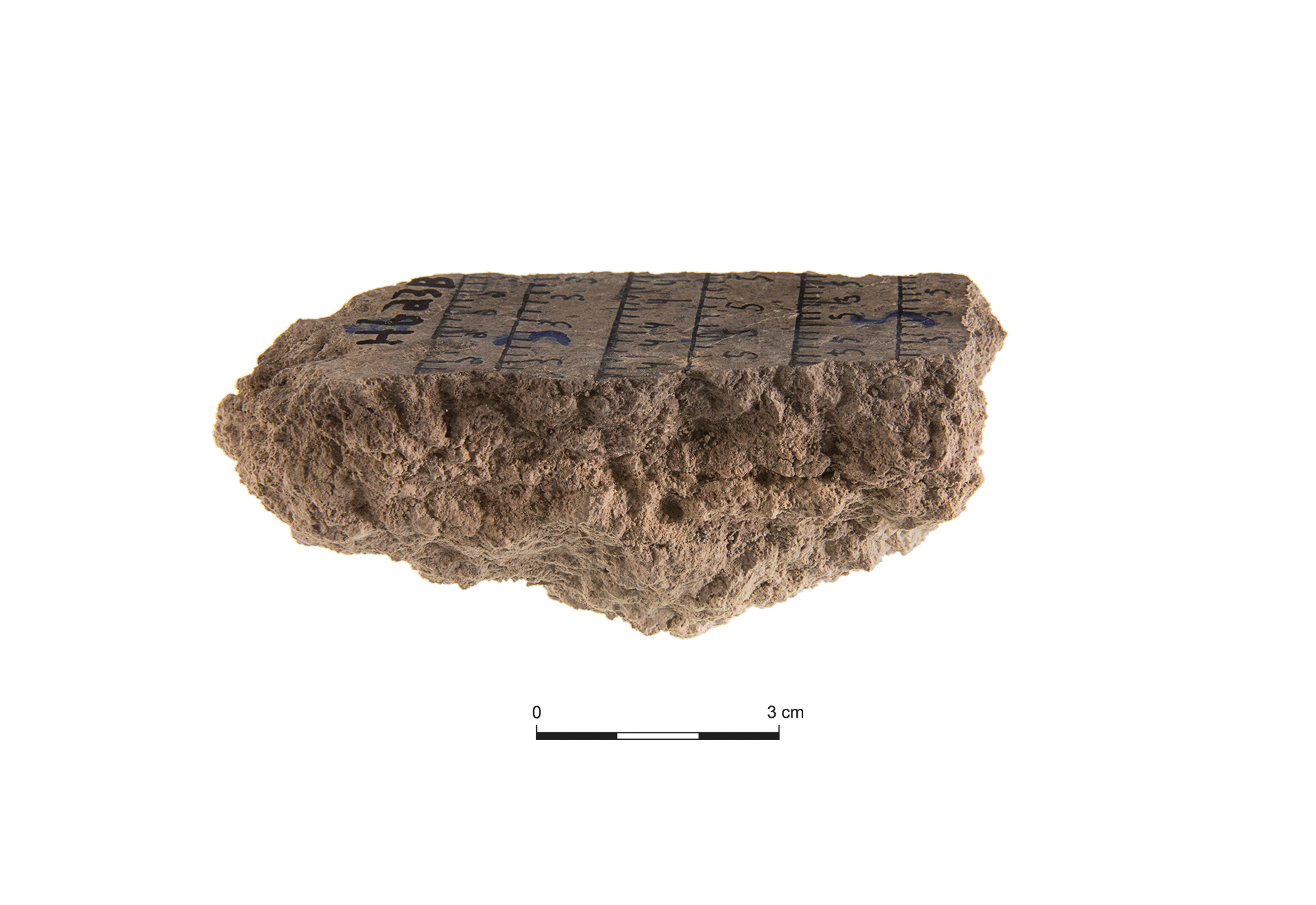

Supplement: S3 Fig — These segments consist of a single layer. Photograph by Sasha Flit. (JPG) [file pone.0237029.s003.jpg]

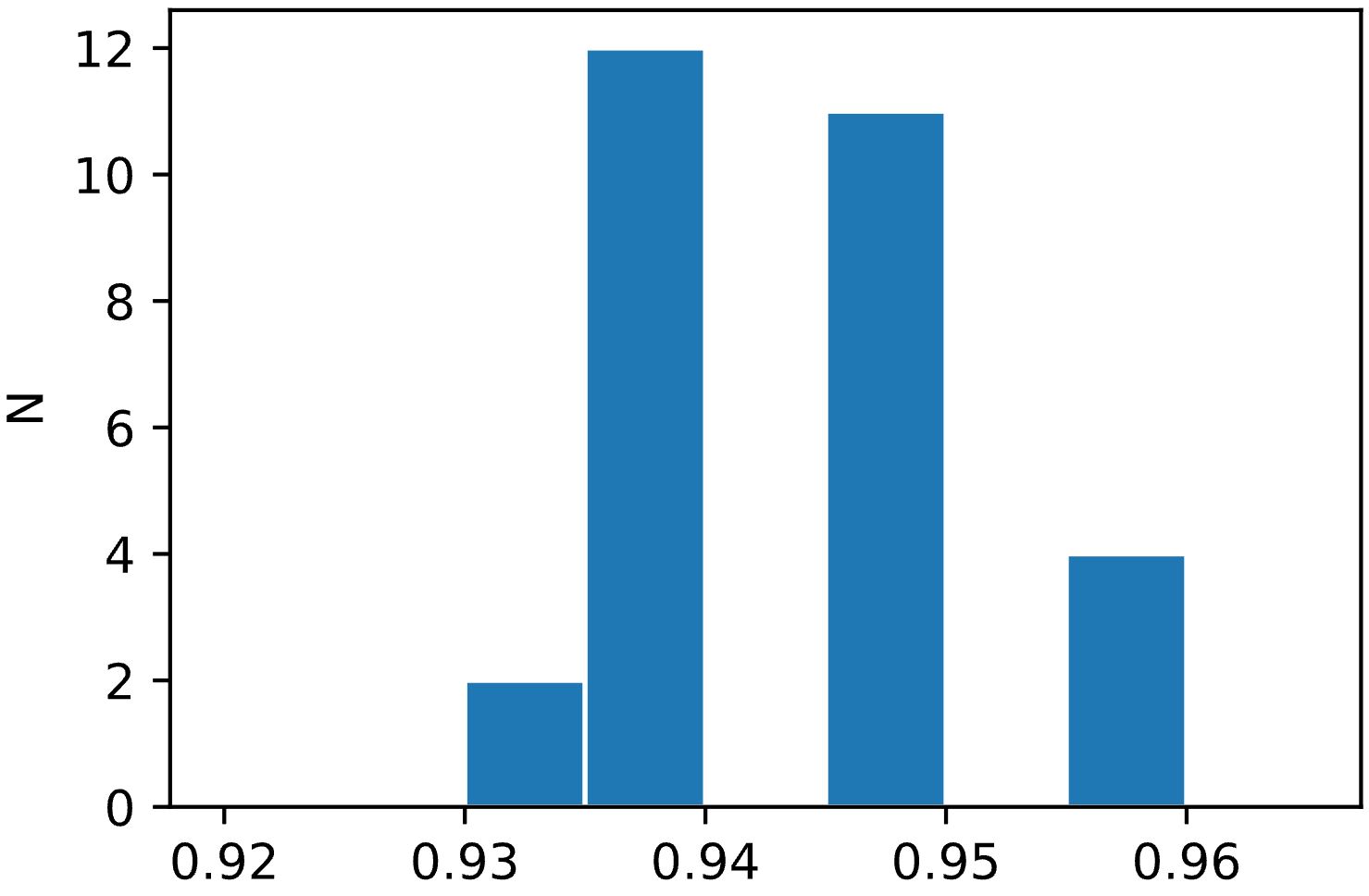

Supplement: S6 Fig — (JPG) [file pone.0237029.s006.JPG]

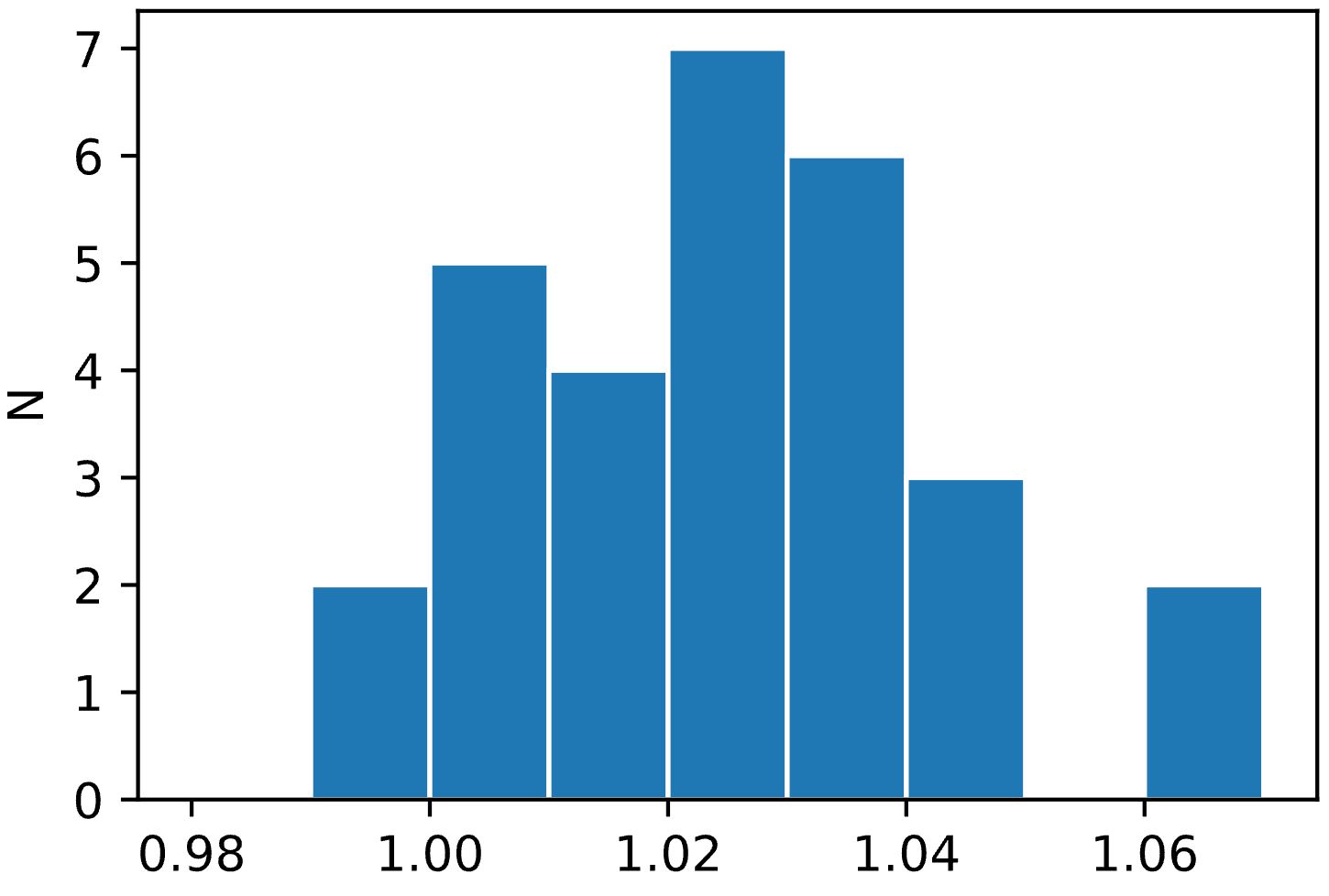

Supplement: S7 Fig — (JPG) [file pone.0237029.s007.JPG]
