# Supplementary material for: The Earth’s magnetic field in Jerusalem during the Babylonian destruction: A unique reference for field behavior and an anchor for archaeomagnetic dating
Source: PLoS One. 2020 Aug 7;15(8):e0237029. doi: 10.1371/journal.pone.0237029 (PMC7413505; doi:10.1371/journal.pone.0237029)
Supplement: S1 Table — (PDF) [file pone.0237029.s010.pdf]

Floor Segment	Basket	Locus	Dip [a]	Declination	Inclination	n[b]	k[c]	α_{95}[c]
HG1A	17620	1356	054,73	8.1	56.7	9	472	2.4
HG1B	17621	1356	094,44	16.2	52.6	6	1104	2
HG1C	17622	1356	090,47	8.3	54.6	6	714	2.5
HG1D	17623	1356	083,42	13	56.8	7	330	3.3
HG1E	17624	1356	095,41	18.8	50	7	305	3.5
HG1F	17625/1	1356	103,53	13.1	53.1	10	972	1.6
HG1G	17625/2	1356	086,35	14.8	54.8	8	254	3.5
HG1H	17626	1356	104,29	28.8	48.3	6	2149	1.4
HG1I	17627/1	1356	104,28	25.1	46	7	1170	1.8
HG1J	17627/2	1356	106,32	22.1	46.7	9	364	2.7
HG1K	17627/3	1356	092,39	9.5	53.4	10	574	2
HG1L	17627/4	1356	108,36	17.2	44.3	9	3274	0.9
HG1M	17627/5	1356	109,37	19.1	46.7	12	405	2.2
HG5A	17981/1	1450	086,38	356.9	67.6	6	822	2.3
HG6A	17981/2	1450	061,72	11.1	61.6	5	1536	2
HG12A	17981/3	1450	329,32	3.7	67.1	6	1336	1.8
HG14A	18294	1481	134,66	16.4	47.2	6	1235	1.9
HG14B	18295	1481	207,62	356.7	45.1	6	1450	1.8
HG14C	18296	1481	184,44	7.2	32.5	12	143	3.6
HG14H	18502	1481	146,51	344.8	54.1	12	98	4.4
HG14J	18504	1481	149,31	352.9	41.4	4	250	5.8
HG14K	18505	1481	173,25	2.1	41.5	4	390	4.7
HG14M	18507	1481	351,6	3.6	57.6	4	963	3
HG14N	18508	1481	147,23	0.3	52	5	370	4
HG14Q	18553	1481	126,29	353	59.4	6	942	2.2
HG18A	18554	1482	235,28	18.2	41.2	6	239	4.3
HG20A	18555	1488	044,19	3.1	52.9	7	766	2.2
HG20B	18556	1488	011,62	6.4	59.4	6	613	2.7
HG22B	18799	1488	358,66	11.4	62.3	6	715	2.5
HG22C	18798	1488	021,74	3.1	61.7	7	514	2.7
HG22D	18797	1488	316,47	1.5	49.6	5	1108	2.3
HG22E	18956	1488	347,29	351.8	62.8	6	670	2.6
HG25A	18950	1503	000,27	354.8	57.5	4	265	5.7
HG25D	18953	1503	031,32	14.5	66	5	396	3.9
HG25F	18955	1488	061,26	351.2	59	5	540	3.3
HG27A	18958	1494	183,20	10.2	39.9	9	697	2
HG28A	18961	1484	203,18	15.6	38.8	10	388	2.5
HG29A	19474	1386	008,79	7.4	57.8	8	546	2.4

[a] Dip direction and dip before declination correction of the magnetic azimuth
[b] Number of specimens used to calculate the mean direction and the statistical parameters
[c] Fisher statistics parameters [1]

1. Fisher RA. Dispersion on a sphere. Proc R Soc Lond A. 1953;217:295-305.
