# Supplementary material for: The Earth’s magnetic field in Jerusalem during the Babylonian destruction: A unique reference for field behavior and an anchor for archaeomagnetic dating
Source: PLoS One. 2020 Aug 7;15(8):e0237029. doi: 10.1371/journal.pone.0237029 (PMC7413505; doi:10.1371/journal.pone.0237029)
Supplement: S2 Table — (PDF) [file pone.0237029.s011.pdf]

Floor Segment	Basket	Locus	Dip	Declination	Inclination	n	k	α_{95}
HG14D	18297	1481	207,63	31.5	3.3	5	324	4.3
HG21A	18795	1488	036,66	178.3	70.4	6	617	2.7
HG21B	18796	1488	036,68	189.6	69.1	5	476	3.5
HG22A	18801	1488	007,80	219.9	74.9	6	432	3.2

* Headers as in Table S1
