# Supplementary material for: The Earth’s magnetic field in Jerusalem during the Babylonian destruction: A unique reference for field behavior and an anchor for archaeomagnetic dating
Source: PLoS One. 2020 Aug 7;15(8):e0237029. doi: 10.1371/journal.pone.0237029 (PMC7413505; doi:10.1371/journal.pone.0237029)
Supplement: S3 Table — (PDF) [file pone.0237029.s012.pdf]

Floor Segment	Basket	Locus	Dip	Declination	Inclination	n	k	α_{95}
HG13A	18292	1482	076,65	227.9	67.6	4	153	7.5
HG14E	18298	1481	139,46	346.5	53.4	7	69	7.3
HG14F	18299	1481	162,64	346.1	35.9	5	103	7.6
HG14G	18501	1481	353,32	25.8	81.3	5	67	9.4
HG14I	18503	1481	162,64	344.1	25.2	7	12	18
HG14L	18506	1481	049,5	359.5	62.4	4	87	9.9
HG14O	18509/1	1481	116,16	11.3	52.7	2	213	17.2
HG14P	18509/2	1481	061,41	333.7	59.1	4	125	8.3
HG24A	18949	1503	043,104	259.8	30.4	3	347	6.6
HG25B	18951	1503	333,29	18.1	54.9	2	1005	7.9
HG25C	18952	1503	333,30	22.7	64.1	5	86	8.3
HG25E	18954	1488	087,21	1.9	55.8	6	43	10.3

* Headers as in Table S1
