# Supplementary material for: The Earth’s magnetic field in Jerusalem during the Babylonian destruction: A unique reference for field behavior and an anchor for archaeomagnetic dating
Source: PLoS One. 2020 Aug 7;15(8):e0237029. doi: 10.1371/journal.pone.0237029 (PMC7413505; doi:10.1371/journal.pone.0237029)
Supplement: S4 Table — (PDF) [file pone.0237029.s013.pdf]

| Floor segment | Specimen | Bmin (μT) | Bmax (μT) | B (μT) | Tmin (ºC) | Tmax (ºC) | Anisotropy correction | Cooling rate correction | FRAC | β | MAD | DANG |
|---|---|---|---|---|---|---|---|---|---|---|---|---|
| HG01A | 12i | 76.7 | 86.6 | 76.7 | 100 | 280 | 1.04 | 0.95 | 0.86 | 0.09 | 1.62 | 1.33 |
| HG01A | 13i | 70.8 | 79.2 | 79.2 | 100 | 260 | 1 | 0.93 | 0.83 | 0.01 | 2.79 | 2.47 |
| HG01B | 06i | 81.4 | 83.2 | 81.4 | 0 | 320 | 1.03 | 0.95 | 0.91 | 0.02 | 3.91 | 1.69 |
| HG01B | 06k | 78.8 | 83.1 | 78.8 | 0 | 320 | 0.99 | 0.95 | 0.91 | 0.03 | 1.72 | 0.66 |
| HG01C | 04i | 76.7 | 82.6 | 77 | 100 | 470 | 1.03 | 0.94 | 0.88 | 0.05 | 2.56 | 0.85 |
| HG01C | 04k | 73 | 79.5 | 77.5 | 0 | 300 | 1 | 0.94 | 0.8 | 0.02 | 3.56 | 1.37 |
| HG01D | 03i | 82.1 | 83.8 | 82.1 | 0 | 320 | 1.02 | 0.95 | 0.92 | 0.02 | 2.41 | 1.23 |
| HG01D | 03k | 77.3 | 82.4 | 77.7 | 0 | 320 | 1.02 | 0.95 | 0.92 | 0.03 | 3.19 | 1.19 |
| HG01E | 03i | 78.6 | 80.7 | 78.6 | 0 | 320 | 1.01 | 0.94 | 0.97 | 0.02 | 3.01 | 0.6 |
| HG01E | 03k | 80.8 | 85.9 | 80.8 | 100 | 320 | 1.03 | 0.94 | 0.86 | 0.02 | 2.28 | 1.24 |
| HG01F | 01i | 76.9 | 83.1 | 78 | 100 | 470 | 1.01 | 0.96 | 0.9 | 0.04 | 1.69 | 0.22 |
| HG01F | 01k | 77.2 | 83.1 | 78.1 | 100 | 470 | 1 | 0.95 | 0.89 | 0.04 | 0.99 | 0.62 |
| HG01G | 20i | 71.8 | 78.6 | 78.5 | 0 | 260 | 1.02 | 0.94 | 0.86 | 0.02 | 2.9 | 2.18 |
| HG01G | 24i | 70 | 74.9 | 74.9 | 0 | 260 | 1.02 | 0.94 | 0.84 | 0.03 | 2.38 | 1.69 |
| HG01H | 06i | 71 | 79.1 | 78.4 | 100 | 280 | 1.01 | 0.94 | 0.9 | 0.02 | 1.37 | 1.17 |
| HG01H | 06k | 69.9 | 77.9 | 77.9 | 100 | 280 | 1.02 | 0.94 | 0.9 | 0.03 | 1.39 | 1.02 |
| HG01I | 03i | 71.3 | 77.3 | 77.3 | 0 | 240 | 1.03 | 0.94 | 0.86 | 0.05 | 2.98 | 1.54 |
| HG01K | 01i | 72.7 | 83.2 | 77.9 | 0 | 260 | 1.03 | 0.93 | 0.82 | 0.06 | 3.11 | 0.89 |
| HG01L | 01i | 75.5 | 84.2 | 76 | 100 | 300 | 1.01 | 0.95 | 0.87 | 0.06 | 1.49 | 1.09 |
| HG01L | 01k | 75.5 | 84.4 | 77.1 | 100 | 300 | 1.02 | 0.95 | 0.9 | 0.06 | 1.19 | 1.62 |
| HG01M | 01i | 74 | 83.6 | 79 | 0 | 260 | 1.02 | 0.95 | 0.91 | 0.04 | 2.79 | 2.04 |
| HG01M | 02i | 75 | 80.5 | 78.7 | 0 | 260 | 1.04 | 0.96 | 0.89 | 0.04 | 4.13 | 2.21 |
| HG06A | 01i | 72.5 | 77.8 | 77.8 | 100 | 320 | 1 | 0.94 | 0.81 | 0.02 | 2 | 1.27 |
| HG18A | 02i | 62.9 | 62.9 | 62.9 | 100 | 260 | 1.07 | 0.94 | 0.83 | 0.07 | 4.76 | 1.96 |
| HG22B | 03k | 74.8 | 79.1 | 79.1 | 0 | 240 | 1 | 0.96 | 0.8 | 0.04 | 3.32 | 8.87 |
| HG22C | 04i | 67.2 | 75.3 | 75.2 | 0 | 220 | 0.99 | 0.95 | 0.87 | 0.03 | 3.34 | 1.77 |
| HG27A | 01i | 76.9 | 80.1 | 77.2 | 100 | 470 | 1.06 | 0.95 | 0.86 | 0.03 | 0.78 | 0.54 |
| HG27A | 01k | 80.6 | 85.5 | 80.6 | 100 | 470 | 1.06 | 0.96 | 0.84 | 0.04 | 1.32 | 0.13 |
| HG29A | 01i | 71.6 | 71.6 | 71.6 | 0 | 240 | 1.03 | 0.94 | 0.79 | 0.08 | 4.07 | 7.79 |
| HG29A | 01k | 71.3 | 77 | 77 | 0 | 220 | 1.04 | 0.94 | 0.81 | 0.06 | 4.13 | 1.97 |

*$B_{min}$ and $B_{max}$ are the lowest and the highest paleointensity values, respectively, meeting criteria. All other columns relate to the interpretation that met criteria and was chosen by the Thellier GUI [1] following the automatic interpretation approach [2, 3]: B is its paleointensity value, FRAC, β, MAD, DANG are its paleointensity statistics [4].
Specimen HG18A02i (marked in red) was considered an outlier.

1. Shaar R, Tauxe L. Thellier GUI: An integrated tool for analyzing paleointensity data from Thellier-type experiments. Geochemistry Geophysics Geosystems. 2013;14:677-92.
2. Shaar R, Tauxe L, Ben-Yosef E, Kassianidou V, Lorentzen B, Feinberg JM, et al. Decadal-scale variations in geomagnetic field intensity from ancient Cypriot slag mounds. Geochemistry Geophysics Geosystems. 2015;DOI: 10.1002/2014GC005455.
3. Shaar R, Tauxe L, Ron H, Ebert Y, Zuckerman S, Finkelstein I, et al. Large geomagnetic field anomalies revealed in Bronze and Iron Age archaeomagentic data from Tel Megiddo and Tel Hazor, Israel. Earth and Planetary Science Letters. 2016;442:173-85.
4. Paterson GA, Tauxe L, Biggin AJ, Shaar R, Jonestrask LC. On improving the selection of Thellier-type paleointensity data. Geochem Geophys Geosyst. 2014;15.
