# Supplementary material for: The Earth’s magnetic field in Jerusalem during the Babylonian destruction: A unique reference for field behavior and an anchor for archaeomagnetic dating
Source: PLoS One. 2020 Aug 7;15(8):e0237029. doi: 10.1371/journal.pone.0237029 (PMC7413505; doi:10.1371/journal.pone.0237029)
Supplement: S5 Table — (PDF) [file pone.0237029.s014.pdf]

Statistic	Threshold	Description	References
FRAC	0.79	Fraction parameter	Shaar and Tauxe (2013)
β	0.1	Scatter parameter	Coe et al (1978),<br>Selkin and Tauxe (2000)
SCAT	True	Scatter parameter	Shaar and Tauxe (2013)
N_{ptrm}	2	Number of pTRM checks	
n	4	Number of data points	
MAD	5	Maximum Angular Deviation of the zero field steps	Kirschvink (1980)
DANG	10	Deviation Angle of the zero field steps	Tauxe and Staudigel (2004)
Alteration check correction	5%	Alteration check in cooling rate and TRM anisotropy experiments	

* for description of the statistics see Paterson et al. [1]

1. Paterson GA, Tauxe L, Biggin AJ, Shaar R, Jonestrask LC. On improving the selection of Thellier-type paleointensity data. Geochem Geophys Geosyst. 2014;15.
